# Five‐in‐One: Simultaneous isolation of multiple major liver cell types from livers of normal and NASH mice

**DOI:** 10.1111/jcmm.16933

**Published:** 2021-09-23

**Authors:** Ye Zhou, Funmilola Adewale, Sun Kim, Qi Su, David Glass, Mark W. Sleeman, Andrew J. Murphy, Xiping Cheng

**Affiliations:** ^1^ Regeneron Pharmaceuticals, Inc. Tarrytown NY USA

**Keywords:** cell isolation, fluorescence‐activated cell sorting, liver, non‐alcoholic steatohepatitis

## Abstract

NASH is a chronic liver disease that affects 3%–6% of individuals and requires urgent therapeutic developments. Isolating the key cell types in the liver is a necessary step towards understanding their function and roles in disease pathogenesis. However, traditional isolation methods through gradient centrifugation can only collect one or a few cell types simultaneously and pose technical difficulties when applied to NASH livers. Taking advantage of identified cell surface markers from liver single‐cell RNAseq, here we established the combination of gradient centrifugation and antibody‐based cell sorting techniques to isolate five key liver cell types (hepatocytes, endothelial cells, stellate cells, macrophages and other immune cells) from a single mouse liver. This method yielded high purity of each cell type from healthy and NASH livers. Our five‐in‐one protocol simultaneously isolates key liver cell types with high purity under normal and NASH conditions, enabling for systematic and accurate exploratory experiments such as RNA sequencing.

## 1 INTRODUCTION

Non‐alcoholic steatohepatitis (NASH) is a chronic liver disease that affects 3%–6% of individuals in the general population and has no available FDA‐approved medicines.^1^ Although the ‘two‐hit’ theory is well acknowledged in the field,^2^ novel pathway discovery in NASH pathogenesis is in high demand. A recent review emphasized the contribution of hepatocyte‐macrophage‐stellate cell crosstalk in fibrogenesis,^3^ and another report recognized hepatic stellate cells (HSCs) as a hub of intracellular signalling via stellakine secretion.^4^ Unfortunately, the detailed signalling within a specific cell type and complex crosstalk among non‐parenchymal cell (NPC) types are still largely unknown. Consequently, further understanding of how different cell types contribute to NASH pathogenesis is urgent for the identification of novel therapeutic targets.

To date, several methods have been established for liver cell subtype isolation, most in normal livers.^5^, ^6^ The key step is density gradient centrifugation after a two‐step collagenase perfusion.^7^ However, due to the density similarity between liver endothelial cells (LECs) and hepatic stellate cells (HSCs), this method alone failed to achieve satisfying purity. Furthermore, due to the physiological and biological changes of diseased liver cells, especially HSCs, the isolation of cells from normal and disease livers using the reported protocol is challenging, and isolated cell types are limited.^8^, ^9^ Fernández‐Iglesias and colleagues described a high‐purity method in isolating hepatocytes, LECs, HSCs and macrophages simultaneously in healthy and cirrhotic rat livers via centrifugation and sequential plating on coated dishes.^10^ This method is applicable for in vitro study but not for experiments geared towards understanding in vivo biology. Currently, no methods have been reported to isolate both parenchymal and non‐parenchymal cell types in NASH livers for in vivo biology study.

Here, utilizing single‐cell RNA sequencing‐based cell surface markers, we describe a technique that can simultaneously isolate five key cell types in one liver from healthy and NASH mouse models with high purity, enabling the accuracy of downstream mechanistic studies within or among cell types.

## 2 METHODS

### 2.1 Animals and NASH models

10‐week‐old male C57BL/6NTac mice purchased from Taconic Biosciences were housed at Regeneron Pharmaceuticals under standard condition. Mice were fed *ad libitum* with standard chow (Purina Laboratory, Rodent diet 5001) or high‐fat high‐fructose diet (HFHFD) (D09100310i; Research Diets Inc.). For NASH models, mice were fed with HFHFD for 25–30 weeks or HFHFD with intraperitoneal injection of CCL4 (Sigma‐Aldrich) at a concentration of 0.2ml/kg twice per week for 10 weeks.^11^ The vehicle group was fed with chow diet and injected with the same volume of corn oil.

### 2.2 Liver perfusion and digestion

Mice were anaesthetized and perfused with the Liver Perfusion Buffer (Gibco) and then digestion buffer (HBSS containing 5 mM CaCl2, 10 mM HEPES and 0.2 mg/ml collagenase I & II (Liberase TM, Roche)). After digestion, the liver was excised and maintained in the Hepatocyte Wash Media (Gibco) on ice.

### 2.3 Isolation of hepatocytes and non‐parenchymal cells

**FIGURE 1:**
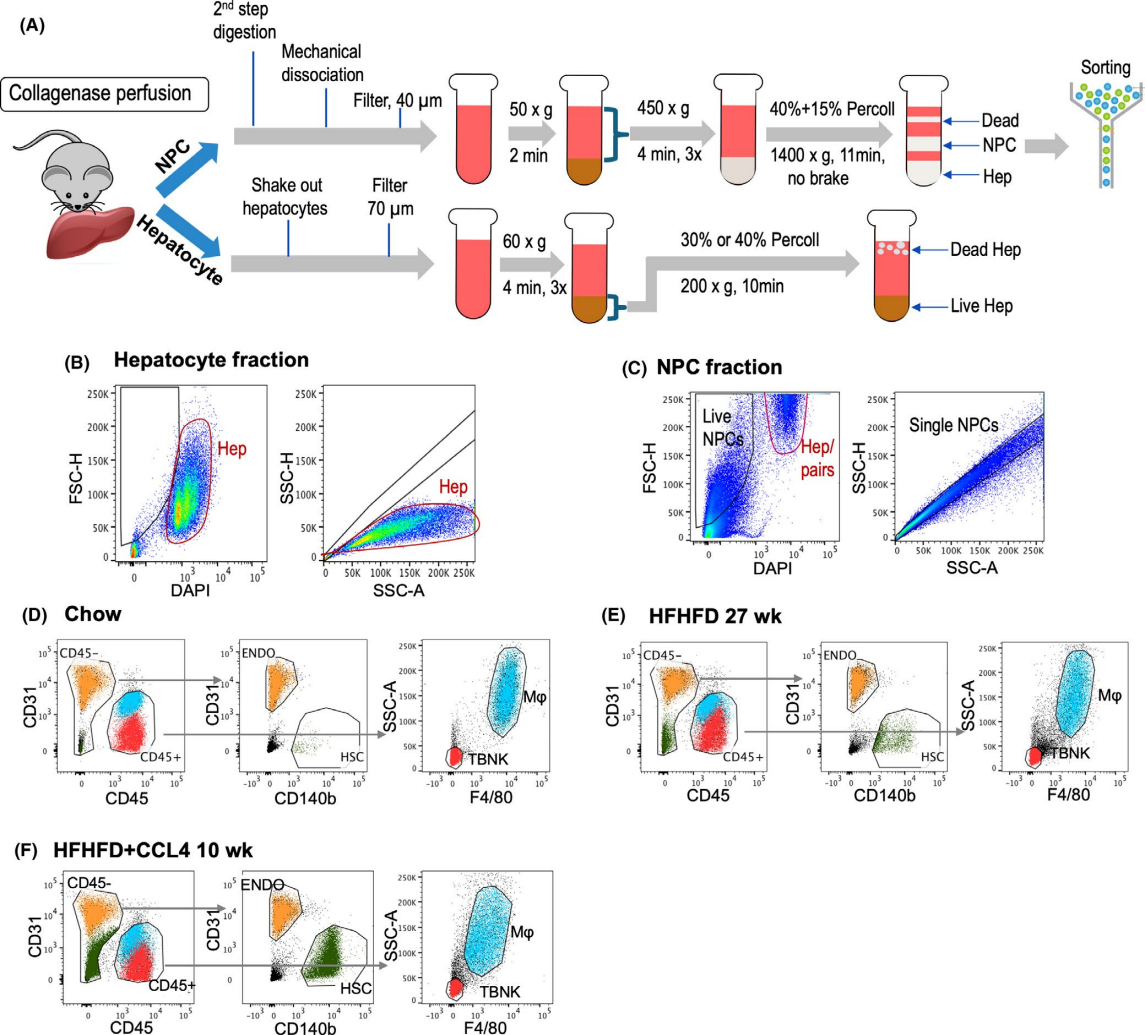
Overview of the workflow and FACS gating strategy. (A) The overview of the workflow. After a two‐step collagenase perfusion, each mouse liver was subsequently split into NPC and hepatocyte fractions to get hepatocytes or NPCs respectively. Liver hepatocytes were collected after Percoll gradient centrifugation. Different NPCs, namely endothelial cells (ENDO), hepatic stellate cells (HSC), macrophages (Mφ) and non‐macrophage immune cells (TBNK), were collected via fluorescence‐activated cell sorting. (B‐F) Gating strategy and FACS panel readouts. (B‐C) Large hepatocytes and hepatocyte‐NPC pairs were recognized and gated out via voltage increase and restrictive gating. Hepatocytes/pairs are circled in red; NPC gating is in black. (D‐F) Gating strategy and panel presentation of chow, HFHFD and HFHFD+CCL4 groups after CD31, CD45, F4/80 and CD140b co‐staining. ENDOs are marked in yellow, macrophages (Mφ) in cyan, TBNK in red and HSCs in green. *n* = 3 independent experiments in each group

Hepatocytes were released from liver lobes and then filtered through 70**‐**μm cell strainers (Corning). After centrifugation and wash, hepatocytes were resuspended in 40% (chow) or 30% (NASH) Percoll (Sigma) in Williams E Media (Gibco) and centrifuged to collect live hepatocytes on the bottom (Figure 1A).

The remaining liver lobes were minced and further digested by 2.5 mg/mL collagenase D (Roche) and 100 ng/ml DNAse I (Sigma) at 37°C for 20–30 min. After centrifugation and wash, NPC pellets were loaded on top of Percoll gradient layers (10mL 15% Percoll on the top and 10mL 40% Percoll on the bottom) for further centrifugation. NPC layer in the middle was collected (Figure 1A).

### 2.4 NPC cell sorting and imaging

NPCs were incubated with Fc‐receptor blocking antibody first, then subsequently stained with antibodies anti‐CD31, anti‐CD45, anti‐F4/80 and anti‐CD140b (Table S1) for sorting. Sorts were performed on BD FACSAria^TM^ Fusion. An unstained hepatocyte sample was run to minimize hepatocyte contamination in the collected fractions.

### 2.5 Statistical analysis

Data were normalized from three independent experiments. All data were analysed by GraphPad Prism software.

For more methods, please refer to Supplementary Material.

## 3 RESULTS

### 3.1 Mice on HFHFD and HFHFD+CCL4 developed NASH phenotype

As shown in Figure S1, the two NASH models developed NASH histology hallmarks, including liver steatosis, inflammation and fibrosis (Figure S1).

### 3.2 Yield and viability

As shown in the FACS plots (Figure 1), different cell types were isolated from both chow and NASH livers. Using this method, we were able to isolate hepatocytes >90% viability from mice on chow and >75% viability from mice on NASH models. The viability of NPCs was consistently >99% across all isolated cell types from chow and NASH models. Cell yields were variable among different cell types (Table S2).

### 3.3 High purity of isolated cells from normal and NASH livers

**FIGURE 2:**
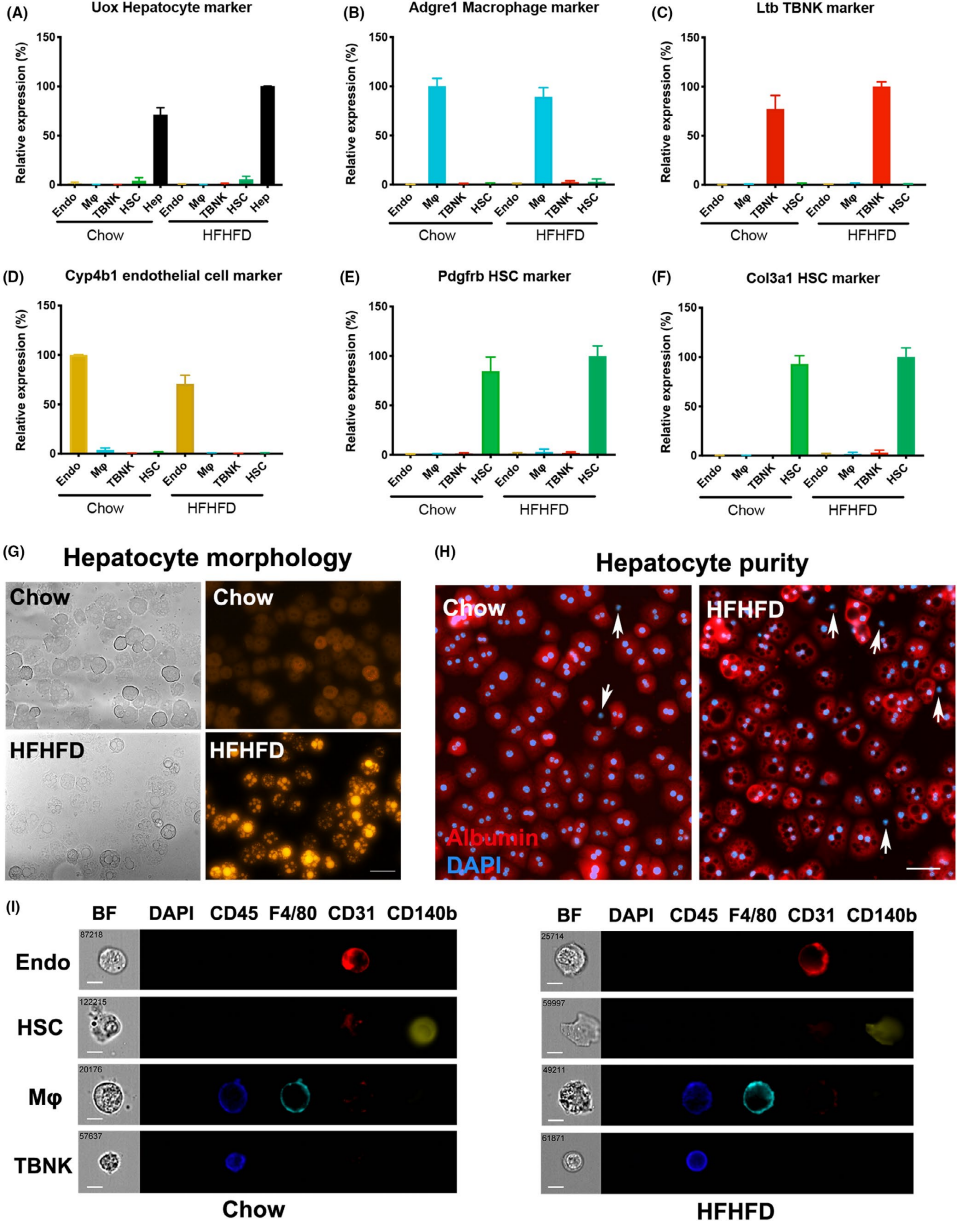
Purity and morphology evaluation on liver cells collected from mice on chow and HFHFD NASH model. Relative gene expression of (A) Uox (Hepatocyte marker), (B) Adgre1 (macrophage marker), (C) Ltb (TBNK marker), (D) Cyp4b1 (endothelial cell markers) and (E) Pdgfrb & (F) Col3a1 (HSC markers) in isolated cell types. Data were normalized by setting the strongest expressed cell type as 100%. Endothelial cells are marked in yellow, macrophages (Mφ) are in cyan, non‐macrophage immune cells (TBNK) are in red, and HSCs are in green. (G) Bright field (left) and Nile Red staining (right) reveal morphology changes and lipid accumulation in steatotic hepatocytes. H, Albumin (red) and DAPI (Blue) staining of primary hepatocytes shows the purity of isolated hepatocytes. Arrows point to non‐hepatocyte cells with negative albumin signals. I, Images of single NPC cells captured from flow cytometry display morphologies and clear cell surface marker staining. Data represent mean ± SEM. *n* = 3 independent experiments. Scale bar is 50 μm in (H) and 7 μm in (I).

To test the purity of yield, we performed gene expression and morphological studies on isolated cells (Figure 2A‐I & Figure S3A‐F). In RT‐PCR, the purity of each cell type was evaluated by the expression of cell type markers identified through scRNA‐seq (Figure S2). We observed an estimate >95% of purity of each cell type. For example, compared to HSCs, non‐HSC cells expressed less than 3.77% and 3.27% of HSC marker CD140b and collagen type III alpha 1 chain (Col3a1) respectively (Figure 2E‐F & Figure S3E‐F). Morphological studies on cultured hepatocytes revealed a pronounced lipid accumulation on hepatocytes isolated from HFHFD mice (Figure 2G). More than 95.1% of normal and 94.1% of steatotic hepatocytes were albumin‐positive (Figure 2H). Images of single NPC cells captured from flow cytometry showed clear cell surface marker staining (Figure 2I).

## 4 DISCUSSION

Though NASH affects 3–6% of individuals in the United States, deep understanding of its pathogenesis is limited. Liver cell type isolation has been achieved by multiple methods, but none of these techniques were applied to NASH livers. Here, we report a Five‐in‐One method that can simultaneously isolate five cell types in healthy and NASH livers.

We could not isolate HSCs by autofluorescence alone or autofluorescence+/CD45‐ (Figure S4), a method suggested by Tacke and Schwabe groups.^8^, ^9^ This is likely due to the disparity of isolation purpose and protocol. Both methods enriched HSCs at the expense of other cell types, while our goal was to retain all NPCs, especially macrophages that emit overwhelming autofluorescence. On top of that, HSCs significantly decrease autofluorescence intensity upon activation in NASH^12^; therefore, we believe cell surface marker‐based, rather than autofluorescence‐based, cell sorting is a better choice for NASH HSC isolation.

Although our method achieved its primary goals, several limitations need to be considered. NPC isolation procedures with enzyme digestion followed by antibody staining and sorting may affect the expression of some genes, rapid signalling events and post‐translational modification process. In addition, this method requires sophisticated skills from the researcher and established platform from the institute, which could be costly regarding labour and equipment expense. Finally, the yield of NPCs, especially HSCs, may not be adequate for downstream applications requiring large amount of material, such as biochemistry studies.

This method requires only a single liver for all five cell types, significantly reducing the number of animals used and assuring an accurate comparison within one animal. The isolated cells can be further processed with RNA analyses and cell‐culture studies for pathogenesis investigation and drug screening. Our method facilitates the opportunity for a deeper understanding of different cell types and their specific signalling pathways, cell‐cell interactions and contributions to pathogenesis, which may lead to the discovery of novel drug targets.

## CONFLICT OF INTEREST

The authors declare no conflicts of interest to disclose. All authors are employees and shareholders of Regeneron Pharmaceuticals.

## AUTHOR CONTRIBUTIONS

**Ye Zhou:** Conceptualization (lead); Data curation (lead); Formal analysis (lead); Investigation (lead); Methodology (lead); Project administration (lead); Writing‐original draft (lead). **Funmilola Adewale:** Conceptualization (supporting); Data curation (equal); Formal analysis (equal); Methodology (equal); Writing‐review & editing (equal). **Sun Kim:** Conceptualization (equal); Methodology (equal); Writing‐review & editing (equal). **Qi Su:** Data curation (equal); Formal analysis (equal); Software (lead); Writing‐review & editing (supporting). **David Glass:** Conceptualization (equal); Project administration (supporting); Supervision (equal); Writing‐review & editing (equal). **Mark W. Sleeman:** Conceptualization (equal); Project administration (equal); Supervision (equal); Writing‐review & editing (equal). **Andrew J. Murphy:** Conceptualization (equal); Project administration (supporting); Supervision (equal). **Xiping Cheng:** Conceptualization (equal); Investigation (equal); Methodology (equal); Project administration (lead); Supervision (lead); Writing‐review & editing (lead).

## Supporting information

Fig S1

Fig S2

Fig S3

Fig S4

Supplementary Material

## Data Availability

The data that support the findings of this study are available from the corresponding author upon reasonable request.
