# Supplementary figures and images for: Five‐in‐One: Simultaneous isolation of multiple major liver cell types from livers of normal and NASH mice
Source: J Cell Mol Med. 2021 Sep 23;25(20):9878–83. doi: 10.1111/jcmm.16933 (PMC8505823; doi:10.1111/jcmm.16933)

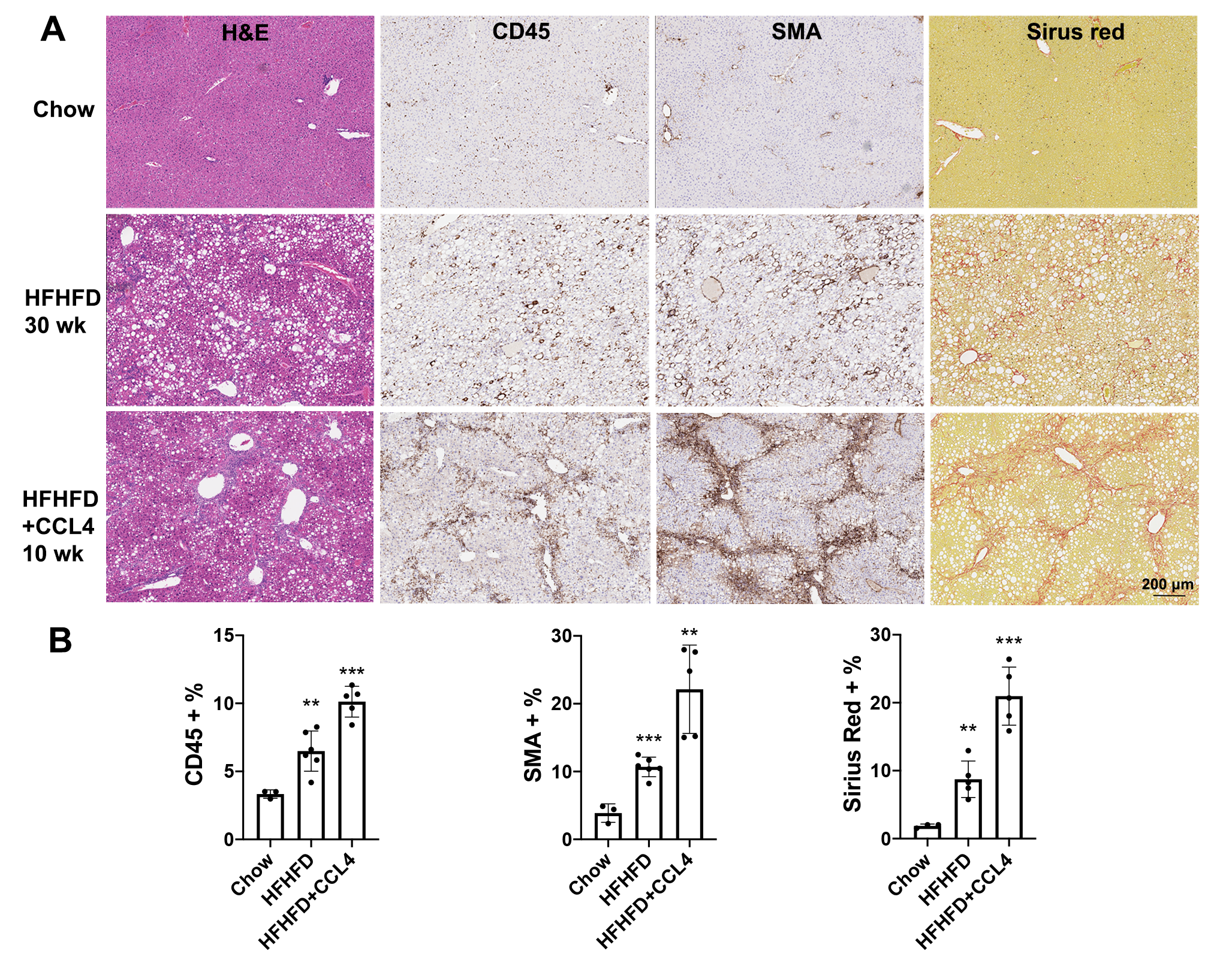

Supplement: Supplementary file 1 — Fig S1 [file JCMM-25-9878-s005.tif]

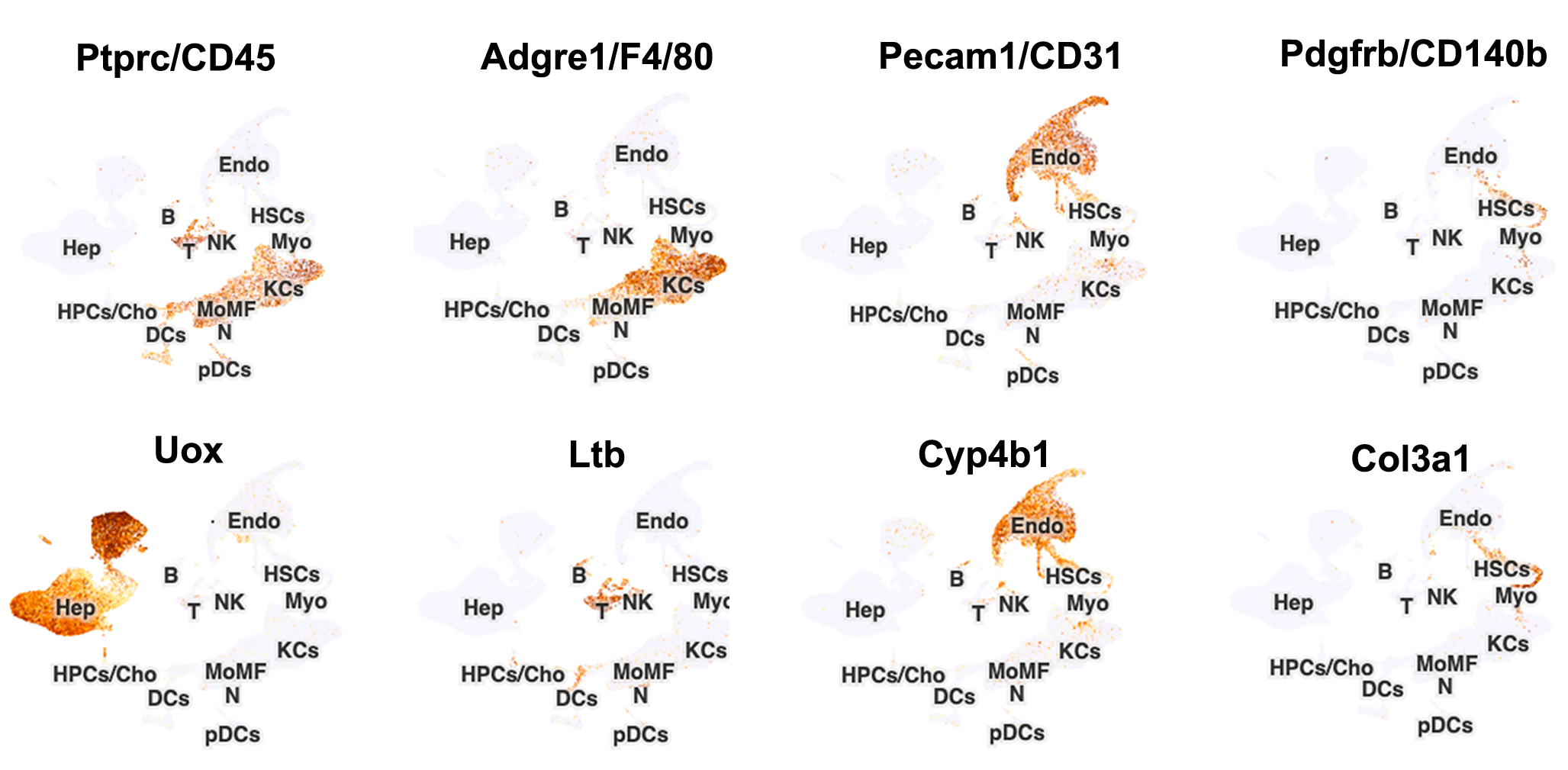

Supplement: Supplementary file 2 — Fig S2 [file JCMM-25-9878-s001.tif]

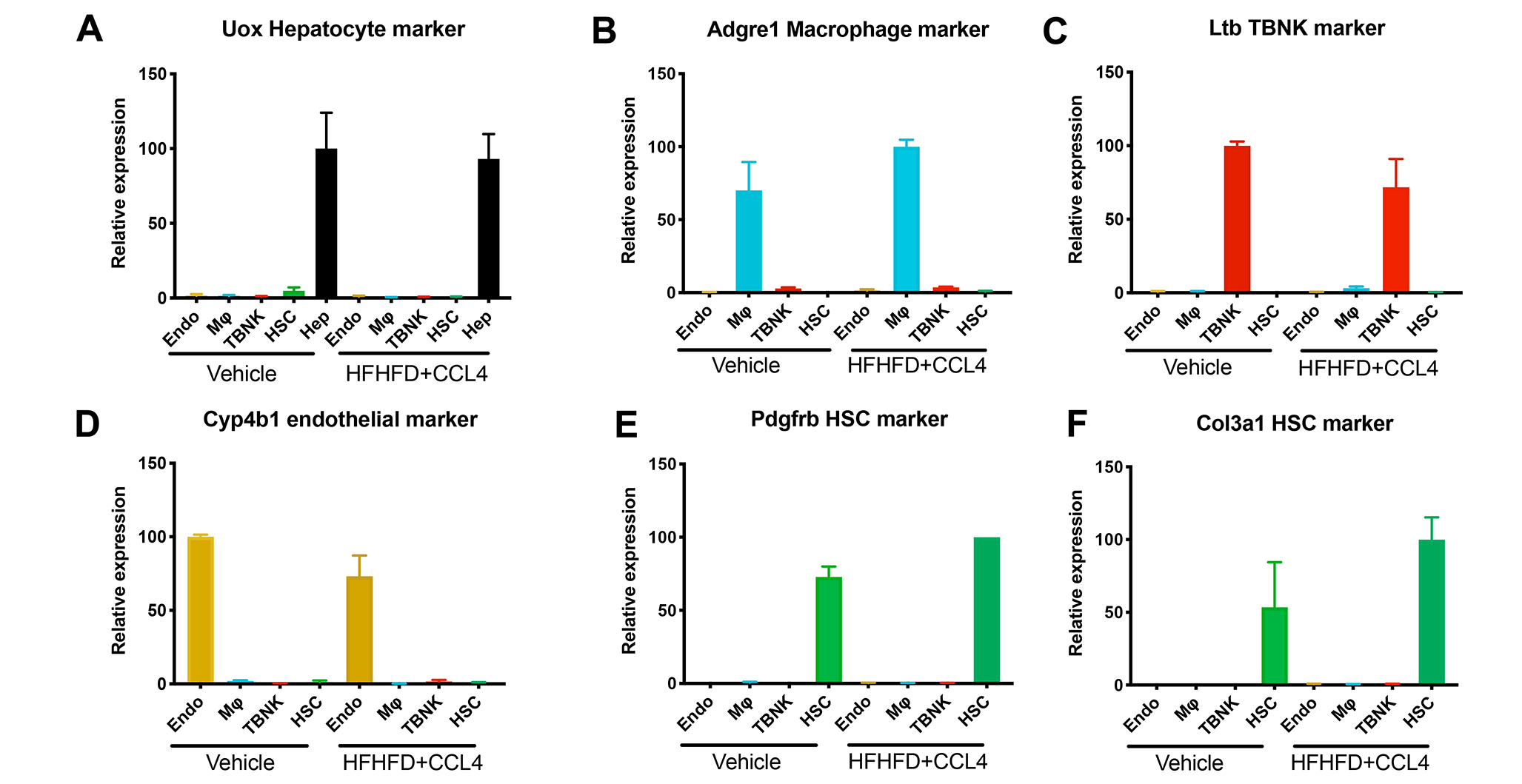

Supplement: Supplementary file 3 — Fig S3 [file JCMM-25-9878-s002.tif]

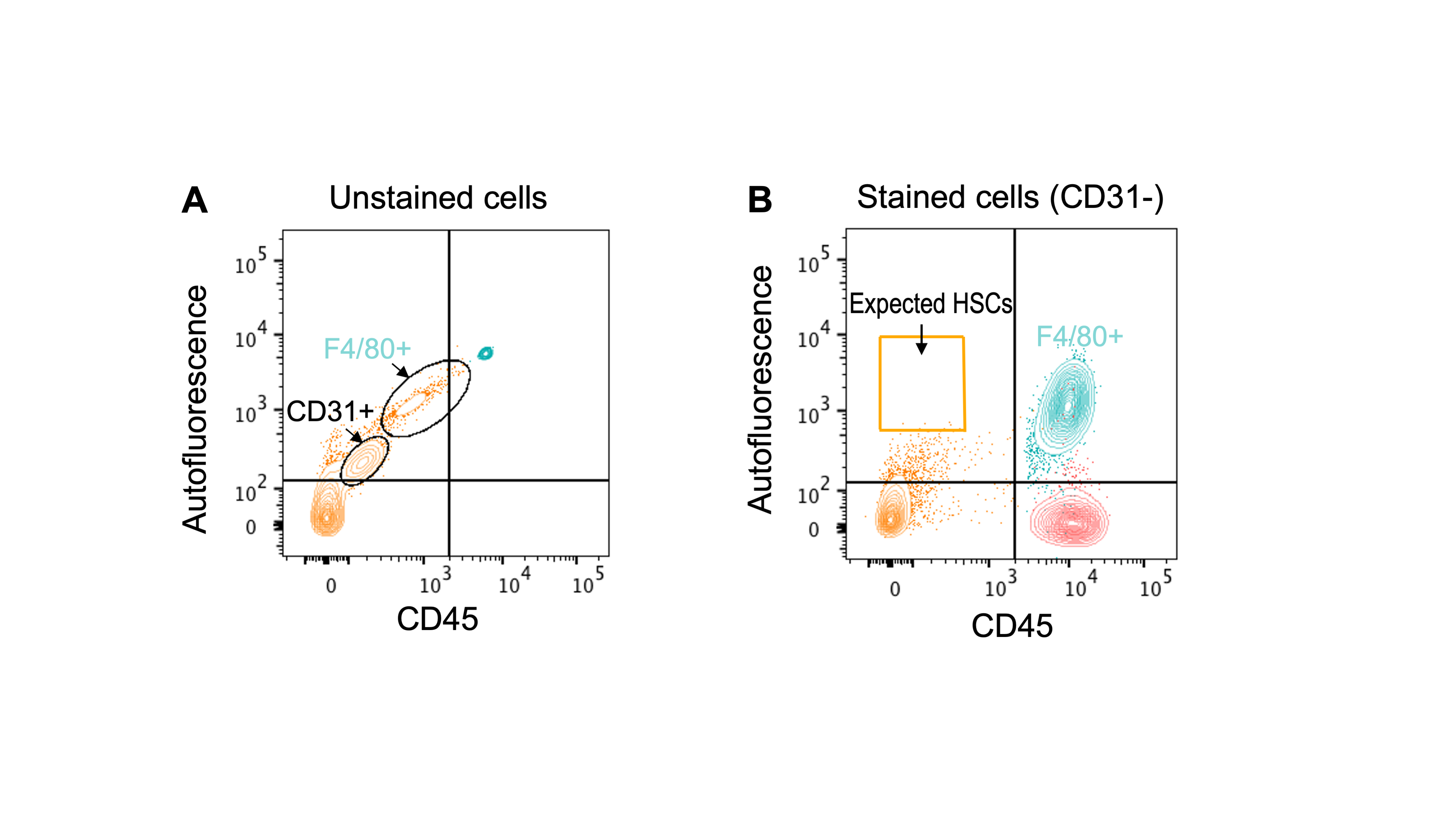

Supplement: Supplementary file 4 — Fig S4 [file JCMM-25-9878-s004.png]
