# Supplementary material for: Five‐in‐One: Simultaneous isolation of multiple major liver cell types from livers of normal and NASH mice
Source: J Cell Mol Med. 2021 Sep 23;25(20):9878–83. doi: 10.1111/jcmm.16933 (PMC8505823; doi:10.1111/jcmm.16933)
Supplement: Supplementary file 5 — Supplementary Material [file JCMM-25-9878-s003.docx]

**Supplementary Material and Methods**

*Liver histology and immunostaining*

Formalin-fixed, paraffin-embedded (FFPE) liver samples were sectioned into 5 μm, followed by deparaffination and rehydration. For hematoxylin and eosion (H&E) staining, slides were stained by Carazzi’s hematoxylin (Sigma), followed by eosin-phloxine (Sigma). Sirius red staining was performed using a Picro-Sirius Red Stain Kit (abcam, #150681). For immunostaining, after a heat-induced antigen retrieval, slides were treated with 3% hydrogen peroxide for 10 min. Subsequently, slides were permeabilized with 3% Tween 80 for 10 min and blocked with 5% normal goat serum for 1 h. Anti-CD45 (Abcam, #ab10558) or anti-alpha smooth muscle actin (α-SMA, Abcam, #ab124964) primary antibodies were applied for 3 h at room temperature. After wash, slides were incubated with anti-rabbit HRP for 1 h and developed using DAB substrate. Slides were counterstained with hematoxylin and hydrated for mounting. Images were scanned using a Zeiss Axio Scan Z1 slide scanner and the images were analyzed using Halo software (Indica Labs).

*Cell count and viability*

Hepatocyte count and viability were determined using the Cellometer Auto 2000 system (Nexcelom Bioscience) or BD FACSAria^TM^ Fusion flow cytometer.

*RT-PCR*

Sorted cell fractions were pelleted and processed to total RNA extraction by RNeasy Mini kit (Qiagen, cat#74104). Total RNA was synthesized to cDNA using an iScript cDNA synthesis kit (Bio-rad, cat#1708891). RT-PCR was performed using SYBR Green reagents (Bio-rad, cat#1725017) and customized primers (Integrated DNA Technologies) in a QuantStudio 6 Flex RT-PCR system (Applied Biosystems). 18s RNA was used as an internal control and the relative gene expression was determined using the 2^–∆∆Ct^ method [1].

*Morphology study of isolated cell types*

For hepatocytes, cells were plated on collagen I-coated chamber slides (Falcon) for 3 h at 37°C. Cells were then stained with Nile Red (Sigma, 1μg/mL) or anti-albumin antibody (Sigma, #A90-134A) with DAPI. Morphology images were captured using Zeiss Axio Observer system. For single-cell images of NPCs, after NPC antibody staining, cells were acquired on the Amnis^®^ ImageStream^®X^ Mk II imaging flow cytometer (Luminex Corporation).

| **Antigen** | **Fluorophore** | **Company** | **Catalog #** | **Dilution** |
| --- | --- | --- | --- | --- |
| CD16/32 | NA | BD Biosciences | 553141 | 1:1000 |
| CD31 | BB700 | BD Biosciences | 566490 | 1:150 |
| CD45 | APC-CY7 | BD Biosciences | 557659 | 1:500 |
| F4/80 | AF647 | BD Biosciences | 565853 | 1:200 |
| CD140b | PE | Biolegend | 36006 | 1:20 |

**Supplementary Table 1. Primary antibody in use for FACS panel.** All antibodies are monoclonal rat anti-mouse IgG.

| **Group** | **cell type** | **Yield (10^3^/g liver)** | **Viability** |
| --- | --- | --- | --- |
| Chow/Vehicle | Hepatocytes | 25,000 | 90%-95% |
|  | Macrophages | 125-180 | >99% |
|  | non-mac immune cells | 90-120 | >99% |
|  | Endothelial cells | 700 | >99% |
|  | Stellate cells | 4.5-6.9 | >99% |
| HFHFD | Hepatocytes | 875 | 75%-85% |
|  | Macrophages | 90-146 | >99% |
|  | non-mac immune cells | 130-160 | >99% |
|  | Endothelial cells | 214 | >99% |
|  | Stellate cells | 3-5 | >99% |
| HFHFD+CCL4 | Hepatocytes | 7,500 | 80%-88% |
|  | Macrophages | 375-600 | >99% |
|  | non-mac immune cells | 350-430 | >99% |
|  | Endothelial cells | 700 | >99% |
|  | Stellate cells | 40-50 | >99% |

**Supplementary Table 2. Yield and viability.** Note that the average liver weights from chow, vehicle, HFHFD and HFHFD+CCL4 mice were 1.71 ± 0.09 g, 1.49 ± 0.10 g, 5.63 ± 0.80 g and 1.57 ± 0.17 g, respectively.

**Reference**

1. Livak KJ, Schmittgen TD: Analysis of relative gene expression data using real-time quantitative PCR and the 2(-Delta Delta C(T)) Method. *Methods* 2001, 25(4):402-408.
